# Non-Line-of-Sight Perception Method for Autonomous Haul Trucks in Open-Pit Mines Based on 4D mmWave Radar and LiDAR Fusion

**DOI:** 10.3390/s26144615

**Published:** 2026-07-21

**Authors:** Jianjian Yang, Yuyu Zhang, Zhiyao Zheng, Yuyuan Zhang

**Affiliations:** 1 Inner Mongolia Research Institute, China University of Mining and Technology-Beijing, Ordos 017004, China; yangjj@cumtb.edu.cn (J.Y.); sqt2400402053@student.cumtb.edu.cn (Z.Z.); zhangyuyuanwangyi@163.com (Y.Z.); 2 School of Mechanical and Electrical Engineering, China University of Mining and Technology-Beijing, Beijing 100083, China; 3 Key Laboratory of Intelligent Mining and Robotics, Ministry of Emergency Management, Beijing 100083, China

**Keywords:** open-pit mine, autonomous mining truck, 4D millimeter-wave radar, LiDAR, non-line-of-sight perception, multimodal fusion, point cloud processing, volume recovery rate

## Abstract

In open-pit mining environments, large equipment frequently causes severe occlusion, creating critical perception blind spots for LiDAR. Meanwhile, 4D millimeter-wave (mmWave) radar provides penetration capability but is highly susceptible to multipath interference generated by metallic structures and uneven terrain. To address these challenges, this paper proposes a Blind-Spot Complementary Fusion (BSCF) framework that integrates 3D LiDAR and 4D mmWave radar through explicit geometric constraints. The proposed framework first suppresses multipath artifacts and performs calibrated spatiotemporal alignment between heterogeneous sensors. It then introduces high-confidence radar observations into LiDAR blind spots through spatial consistency verification, providing existence-level hidden-target risk cues under extreme occlusion. In addition, a Volume Recovery Rate (VRR) proxy metric is proposed to quantitatively describe envelope-level spatial evidence in occluded regions. Experiments conducted on real-world mining datasets demonstrate that the proposed method effectively suppresses severe multipath interference and improves scene-level cross-modal proximity by approximately 16.5%, while the overlap-region RMSE remains approximately 0.18 m. Under complete occlusion where LiDAR observations are unavailable, the framework provides target-existence risk cues with a VRR of up to 15.6%, providing a useful preliminary indication of hidden target existence. Ultimately, the proposed approach enhances perception robustness and safety for autonomous transportation systems in open-pit mines.

## 1. Introduction

In open-pit autonomous transportation systems, the completeness and high reliability of environmental perception are critical prerequisites for ensuring operational safety. However, in typical operational scenarios such as discharge areas, there are usually numerous large-scale obstacles, including multiple mining trucks parked in parallel or interleaved, fluctuating piles of earth and rock, large-area slopes, and retaining walls. These physical barriers create complex occlusion conditions that obstruct the direct line-of-sight (LOS) of traditional on-board sensors, significantly increasing perception uncertainty.

Currently, Light Detection and Ranging (LiDAR) has become the primary sensor for environment perception in autonomous mining due to its high resolution and dense 3D point cloud data. However, LiDAR relies on the linear emission and reflection of infrared beams, making it unable to penetrate solid obstacles. Under complex occlusion, extensive blind spots occur within the LiDAR’s scanning field; particularly in close-range occlusion scenarios, a single-LiDAR solution can easily lead to the complete missed detection of hidden targets. This data gap prevents planning and control modules from identifying hidden risks, which may trigger collisions.

Recently, 4D millimeter-wave radar has gained attention for its longer electromagnetic wavelength, exhibiting superior non-line-of-sight (NLOS) detection potential through multipath diffraction and penetration characteristics. This capability offers a new pathway for detecting the existence of hidden obstacles behind foreground occlusions. However, in mining environments, electromagnetic waves undergo severe multipath reflections between giant metallic truck chassis and unstructured ground, causing the radar point cloud to be mixed with substantial multipath ghost targets beneath the surface, severely interfering with accurate target extraction. Meanwhile, most existing perception research remains urban-centric, utilizing datasets like KITTI and nuScenes that are difficult to transfer directly to the harsh physical conditions of open-pit mines. Although specialized mining datasets like AutoMine have recently emerged, they primarily focus on LOS perception and lack multimodal data targeting NLOS conditions in open-pit mines.

At present, blind-spot completion and occlusion reasoning mechanisms for hidden obstacles remain insufficiently explored. Current deep-learning-based Bird’s-Eye-View (BEV) or late-fusion strategies usually implicitly assume that targets can be effectively observed by each modality. In extreme mining scenarios, when a single-modality sensor suffers from severe physical occlusion while the other retains sparse signals, pure feature interaction lacking physical spatial guidance tends to exhibit instability. Table 1 summarizes the limitations of existing methods and identifies the research gap addressed in this study.

Beyond the broad method categories in Table 1, the proposed BSCF framework is positioned as a training-free geometric risk-cue extraction pipeline for mining NLOS scenes, rather than as a directly benchmarked replacement for modern learning-based multimodal fusion frameworks. Its role is to use calibrated LiDAR–radar point clouds and physical blind-zone constraints to complement LiDAR perception under asymmetric visibility, particularly when complete annotated datasets for open-pit NLOS scenarios are unavailable.

To bridge this research gap, this study develops a multimodal NLOS perception method based on physical spatial constraints, utilizing the penetrative characteristics of 4D millimeter-wave radar to selectively introduce risk cues into LiDAR blind regions. The main contributions of this study are summarized as follows:

1. **An explicit geometric constraint-based Blind-Spot Complementary Fusion (BSCF) framework is proposed for non-line-of-sight perception in open-pit mining environments.** By selectively introducing high-confidence radar observations into LiDAR physical blind spots through spatial consistency verification, the proposed framework effectively exploits the complementary sensing characteristics of LiDAR and 4D mmWave radar, thereby improving existence-level hidden-target risk-cue extraction under extreme asymmetric visibility conditions.
2. **A PatchWork-Mine-based traversable-area segmentation strategy is developed for unstructured mining environments.** By reducing the influence of residual multipath-induced geometric distortions on local plane fitting, the proposed method achieves robust ground segmentation and reliable foreground obstacle extraction from fused point clouds under complex terrain conditions, providing a high-purity geometric baseline for the subsequent evaluation of fusion results.
3. **A Volume Recovery Rate (VRR) proxy metric is introduced to quantify envelope-level spatial evidence within occluded regions.** In the investigated NLOS mining scenarios, VRR is used as a main envelope-level indicator of spatial recovery under extreme occlusion, rather than as a full-object reconstruction or semantic detection metric.
4. **The proposed framework is validated using real-world multi-modal data collected from autonomous haulage scenarios at the Haerwusu Open-Pit Coal Mine.** Experimental results demonstrate that the proposed method effectively improves hidden-target risk-cue extraction while maintaining geometric consistency under complex unstructured terrain and extreme occlusion conditions, indicating its potential utility for autonomous mining systems.

## 2. Related Work

### 2.1. NLOS Perception and Multipath Mechanisms

Non-line-of-sight (NLOS) perception aims to maintain environmental awareness when direct line-of-sight observations are unavailable. Compared with optical sensors, millimeter-wave (mmWave) radar exhibits stronger penetration and diffraction capabilities, making it more robust under adverse conditions such as dust, fog, and occlusion [1,2]. However, the effectiveness of radar-based NLOS perception largely depends on the modeling and utilization of multipath propagation.

Recent studies have begun to explore the potential of multipath propagation for NLOS perception. Lai et al. demonstrated the feasibility of radar-based NLOS reconstruction by recovering hidden objects from indirect radar reflections [3]. Ding et al. further exploited multipath propagation for target localization in dense clutter environments, demonstrating that reflected radar signals can provide additional geometric information beyond direct line-of-sight observations [4]. These studies indicate that multipath propagation should not be regarded solely as a source of interference. When properly modeled and utilized, it can also provide valuable information for hidden-target perception and NLOS scene understanding.

Nevertheless, existing approaches are primarily designed for structured indoor environments or urban roads. In open-pit mines, large metallic equipment, irregular terrain, and highly reflective surfaces generate complex propagation paths that significantly increase the occurrence of multipath interference and ghost targets. Consequently, robust methods for suppressing multipath artifacts and extracting useful NLOS information in mining environments remain insufficiently explored.

### 2.2. Multi-Modal Fusion Under Occlusion

To compensate for the limitations of individual sensors, multi-modal fusion has become a mainstream perception paradigm. Existing studies indicate that although 4D mmWave radar provides strong robustness under adverse environmental conditions, its sparse measurements and multipath uncertainty still require complementary geometric information from other sensors such as LiDAR or cameras [5,6,7].

Feature-level fusion methods directly integrate heterogeneous sensor information during feature extraction. Representative approaches such as PointPillars [8], MVX-Net [9], and InterFusion [10] establish cross-modal interactions through voxelized representations and shared feature spaces. These methods effectively improve perception performance when sufficient observations are available.

Decision-level fusion methods combine independent detection results generated by different sensors. CenterFusion [11] associates radar measurements with object proposals from a primary detector, improving deployment flexibility and sensor compatibility. However, their performance remains dependent on the availability of visible object observations.

To improve robustness under complex conditions, recent studies have introduced unified spatial representations, geometric reasoning, and uncertainty-aware perception mechanisms. State-of-the-art frameworks such as BEVFusion [12], FusionBEV [13], MLF-4DRCNet [14], and MSSF [15] enhance multi-modal perception through diverse integration strategies, ranging from unified bird’s-eye-view representations to voxel-level geometric consistency and hierarchical feature interaction. Furthermore, CaDDN [16] incorporates geometric reasoning through depth distribution estimation, while uncertainty-aware evidential fusion frameworks [17] explicitly model sensor uncertainty. Occupancy-based approaches such as SurroundOcc [18] and VoxFormer [19] further attempt to infer hidden structures through occupancy completion and free-space reasoning.

Despite these advances, most existing fusion frameworks implicitly assume that at least partial observations of the target remain available in one or more sensing modalities. Under severe asymmetric visibility conditions commonly encountered in open-pit mines, where LiDAR observations may be completely blocked and radar measurements are extremely sparse and noisy, the effectiveness of conventional feature alignment and occupancy reasoning methods can be substantially degraded.

### 2.3. Mining Perception and Unstructured Terrain Processing

Compared with structured urban roads, autonomous perception in open-pit mines faces significant challenges arising from unstructured terrain, heavy dust, large metallic equipment, and dynamically changing road conditions [20,21].

Recent studies have explored traversability estimation and terrain understanding in off-road environments. RoadRunner [22] learns terrain traversability from multi-modal observations, while UFO [23] incorporates uncertainty-aware LiDAR-image fusion for off-road semantic terrain mapping. These studies highlight the importance of robust terrain modeling in unstructured environments and provide useful insights for mining perception systems.

In addition, Patchwork [24] and Patchwork++ [25] introduced efficient region-wise ground segmentation strategies for rough and layered terrain, while GndNet [26] demonstrated the feasibility of learning-based real-time ground estimation. These methods have achieved promising performance in off-road and unstructured environments.

However, under severe multipath interference, subterranean radar ghost targets can introduce downward distortion in local plane fitting and corrupt geometric references used for subsequent perception tasks. Once the estimated ground plane becomes unreliable, errors may propagate into target clustering, obstacle extraction, and fusion processes. Therefore, maintaining reliable geometric constraints under severe multipath conditions remains a challenging problem in mining perception systems.

In summary, while existing studies have achieved substantial progress in NLOS perception and multi-modal fusion, effective low-level suppression mechanisms for severe multipath interference and explicit blind-spot completion strategies under extreme asymmetric visibility conditions remain insufficiently explored in open-pit mining scenarios. Therefore, the novelty of this work lies primarily in the scenario-specific system design: it investigates NLOS risk-cue extraction in unstructured open-pit mining scenes, where large metallic haul trucks and non-planar terrain cause strong asymmetric visibility, and limited annotated data constrain large-scale supervised benchmarking. The proposed pipeline adopts reliable training-free geometric modules to establish a reproducible baseline for blind-region radar cue extraction in this scenario.

## 3. Methodology

This section details the proposed spatial-geometry-constrained multimodal perception framework designed for extreme occlusion conditions in open-pit mines. Driven by the core mechanism of Blind-Spot Complementary Fusion (BSCF), the framework aims to establish a complete perception pipeline from low-level data preprocessing to high-level structured mapping. It effectively addresses the challenges of high-order multipath interference and asymmetric visibility induced by complex metallic equipment, making it particularly suitable for unloading zones characterized by severe multi-obstacle occlusion.

### 3.1. System Overview

**Figure 1:**
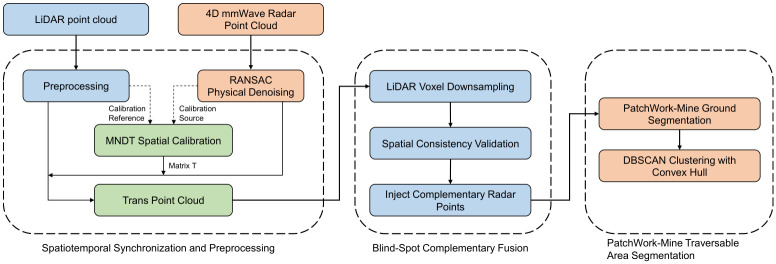
System architecture.

The overall architecture of the proposed BSCF-based perception pipeline is shown in Figure 1.

To construct a more informative environmental perception result under extreme occlusion, the proposed framework identifies the perception blind spots of LiDAR via spatial geometric features and selectively introduces mmWave radar points that have undergone strict spatial morphological filtering. The overall pipeline consists of four main stages:

1. **Spatiotemporal Synchronization and Preprocessing:** Raw data are temporally synchronized via exact timestamps and spatially aligned across different coordinate planes (with the mmWave radar on the XOY plane and LiDAR on the YOZ plane) using the Modified Normal Distributions Transform (MNDT). Subsequently, a RANSAC-based ground-plane geometric constraint model is applied to suppress subterranean multipath ghost targets.
2. **Blind-Spot Complementary Fusion (BSCF):** LiDAR point clouds are voxel-downsampled to build a KD-Tree. Radar points then undergo a two-stage spatial consistency mechanism—first-order spatial consistency validation based on local density and second-order adaptive blind-zone determination—to selectively introduce candidate non-line-of-sight (NLOS) cue points into LiDAR blind zones.
3. **Ground Segmentation:** An improved algorithm, PatchWork-Mine, is adopted to extract drivable areas in unstructured terrain. Based on the Concentric Zone Model (CZM) and Modified Region-wise Ground Plane Fitting (MR-GPF), it incorporates normal uprightness and local flatness constraints to alleviate under-segmentation on undulating surfaces.
4. **Clustering and Feature Extraction:** DBSCAN is employed for distance-based spatial clustering of obstacles, and 3D convex hull volumes are calculated to construct structured grid maps for downstream planning tasks. DBSCAN is used as a conventional instance extraction step, allowing the evaluation to focus on fusion, blind-region cue extraction, and mine-scene traversable-area segmentation.

### 3.2. Spatiotemporal Synchronization and Preprocessing

Under complex occlusion conditions in the unloading zones of open-pit mines, the line-of-sight of LiDAR is often blocked, while the mmWave radar point cloud is highly susceptible to multipath reflections and clutter interference. Therefore, highly accurate spatiotemporal alignment and strict data preprocessing of the raw multi-source perception data are particularly crucial for recovering occluded geometric information and achieving reliable multimodal fusion perception.

First, regarding temporal synchronization, the system adopts a soft synchronization strategy based on exact timestamp alignment, unifying the discrete data streams from heterogeneous sensors under a consistent time reference.

Second, spatial synchronization aims to precisely align cross-modal spatial geometric information. To overcome the three-dimensional perspective differences between the heterogeneous sensors, the system utilizes the Modified Normal Distributions Transform (MNDT) algorithm to calculate a precise rigid homogeneous transformation matrix. This operation accurately maps the non-line-of-sight (NLOS) point cloud of the mmWave radar into the local coordinate system of the LiDAR, establishing a unified spatial reference frame for point cloud fusion.

Finally, following the fundamental coordinate alignment, targeted spatial filtering must be applied to the radar data. The raw 4D mmWave radar point cloud contains a large number of multipath ghost targets caused by multipath reflections from the ground and giant metallic equipment. The relative heights of these false echoes often exhibit significant negative values, which severely disrupts the subsequent blind-spot spatial completion mechanism. Therefore, the preprocessing stage introduces a geometric filtering mechanism based on an improved RANSAC plane fitting algorithm. By robustly estimating the physical ground plane and removing the false echo points located beneath it, this mechanism provides a highly geometrically consistent point cloud input for the subsequent fusion perception.

### 3.3. Blind-Spot Complementary Fusion (BSCF) Algorithm

The core of the BSCF strategy is not to perform direct concatenation of all raw data, but to execute selective point injection driven by explicit spatial geometric constraints.

#### 3.3.1. Mathematical Formulation

Let the preprocessed mmWave radar point cloud be denoted as $P_{R}$ and the LiDAR point cloud as $P_{L}$. To improve computational efficiency, $P_{L}$ is first downsampled using a voxel grid filter, which significantly reduces the point cloud density while preserving the original geometric topology. Subsequently, a KD-Tree data structure is constructed on the downsampled $P_{L}$.

The first stage is **Spatial Consistency Validation**. True obstacle reflection points typically exhibit local spatial aggregation, whereas random noise tends to be discrete. For each radar point $p_{i}\in P_{R}$, a spherical neighborhood search is performed within a radius $r_{n}$. The point is retained as a valid point $p_{i}^{\mathrm{valid}}$ only if the number of spatial neighbors is no less than the predefined threshold $N_{\min}$; otherwise, it is directly discarded as noise:

(1) $$P_{R}^{\mathrm{valid}}=\left\{p_{i}\in P_{R}|\left|N(p_{i},r_{n})\right|\geq N_{\min}\right\},$$

where $N(p_{i},r_{n})$ denotes the set of radar points within the sphere of radius $r_{n}$ centered at $p_{i}$, and the absolute value symbol |·| denotes the cardinality (number of elements) of this set. In the MATLAB implementation used in this study, $N_{\min}$ is set to 3, which requires a radar return to be supported by at least two neighboring radar points rather than appearing as an isolated single-point reflection.

The second stage is **Blind-Zone Determination and Label Fusion**. For each validated radar point $p_{i}^{\mathrm{valid}}$, its nearest neighbor $q_{j}$ in the LiDAR point cloud $P_{L}$ is queried via the KD-Tree. The Euclidean distance $d_{\min}$ between them is then computed:

(2) $$d_{\min}\left(p_{i}^{\mathrm{valid}},P_{L}\right)=\min_{q_{j}\in P_{L}}{∥p_{i}^{\mathrm{valid}}-q_{j}∥}_{2}.$$

If $d_{\min}>r_{\mathrm{blind}}$, it physically indicates that the LiDAR lacks effective observation in this region. The radar point is thus deemed to be located in a perception blind spot and is marked as a “complementary point” to be injected into the fusion space. Conversely, if the distance is less than or equal to the threshold, the area is considered sufficiently covered by the LiDAR, and the radar point is discarded as overlapping redundant data. Finally, the system outputs the fused point cloud and attaches an independent modality source label to each point, thereby preserving the confidence priors that distinguish sensor characteristics for downstream perception tasks. The blind-zone threshold $r_{\mathrm{blind}}$ is set to 0.3 m in the experiments. This value is chosen according to the LiDAR-supported spatial resolution after voxel downsampling and the overlap-region calibration diagnostic, where jointly observable radar points show a post-calibration nearest-neighbor RMSE of approximately 0.18 m. Therefore, $r_{\mathrm{blind}}$ is larger than the residual cross-modal alignment fluctuation in visible regions and is used to separate LiDAR-supported regions from LiDAR-missing blind regions.

#### 3.3.2. BSCF Algorithm Formalization

The complete execution pipeline of the proposed spatial-geometry-constrained fusion mechanism is summarized in Algorithm 1. By incorporating an explicit label assignment process, the algorithm effectively preserves the confidence priors and modality characteristics of different sensors while achieving spatial complementarity.
**Algorithm 1** Blind-Spot Complementary Fusion (BSCF)**Require:** LiDAR point cloud $P_{L}$, Spatially synchronized and preprocessed mmWave radar point cloud $P_{R}$;Voxel size $v_{s}$, Search radius $r_{n}$, Min neighbors $N_{\min}$, Blind-zone threshold $r_{\mathrm{blind}}$.**Ensure:** Fused point cloud with modality source labels $P_{fused}$.  1:$P_{L}^{down}\leftarrow \mathrm{VoxelDownsample}\left(P_{L},v_{s}\right)$;  2:$T_{L}\leftarrow \mathrm{BuildKDTree}\left(P_{L}^{down}\right)$;  3:$P_{fused}\leftarrow \mathrm{AssignLabel}\left(P_{L}^{down},l_{LiDAR}\right)$;    *// Assign modality label to points*  4:$P_{R}^{\mathrm{valid}}\leftarrow \emptyset$;  5:/* Stage 1: Spatial Consistency Validation */  6:**for** each point $p_{i}\in P_{R}$ **do**  7:    **if** $|\mathrm{SearchNeighbors}\left(p_{i},P_{R},r_{n}\right)|\geq N_{\min}$ **then**  8:       $P_{R}^{\mathrm{valid}}\leftarrow P_{R}^{\mathrm{valid}}\cup \left\{p_{i}\right\}$;  9:    **end if**10:**end for**11:/* Stage 2: Blind-Zone Determination and Label Fusion */12:**for** each point $p_{i}\in P_{R}^{\mathrm{valid}}$ **do**13:    $q_{j}\leftarrow \mathrm{FindNearestNeighbor}\left(T_{L},p_{i}\right)$;14:    $d_{\min}\leftarrow {∥p_{i}-q_{j}∥}_{2}$;15:    **if** $d_{\min}>r_{\mathrm{blind}}$ **then**16:       $P_{fused}\leftarrow P_{fused}\cup \left\{\mathrm{AssignLabel}\left(p_{i},l_{Radar}\right)\right\}$    *// Inject complementary point*17:    **end if**18:**end for**19:**return** $P_{fused}$;

### 3.4. PatchWork-Mine Traversable Area Segmentation

To extract traversable free space in open-pit mines, a ground segmentation algorithm adapted to mining terrain, termed PatchWork-Mine, is utilized. Building upon the classical Patchwork algorithm, it integrates the Concentric Zone Model (CZM) and Modified Region-wise Ground Plane Fitting (MR-GPF). It is specifically extended and adapted for the unique unstructured terrain and sensor noise characteristics of mining areas to accommodate unstructured undulating topographies and mitigate the interference of residual multipath noise.

#### 3.4.1. Algorithm Logic and Mathematical Derivation

Initially, the algorithm performs fundamental data preprocessing on the input fused point cloud, filtering out invalid coordinate points containing Not-a-Number (NaN) or infinite (Inf) values to ensure the stability of subsequent matrix operations. If the number of valid points after cleaning fails to meet the system’s predefined minimum threshold, it directly concludes that there is no ground point cloud and terminates the computation for the current frame.

Following validation, the preprocessed point cloud space is divided into $N_{z}$ concentric annular zones. Each zone $Z_{m}\left(m=1,\ldots ,N_{z}\right)$ is defined by a minimum radius $L_{m}^{\min}$ and a maximum radius $L_{m}^{\max}$. For any point $p_{i}\left(x_{i},y_{i},z_{i}\right)$, its radial distance $r_{i}=\sqrt{x_{i}^{2}+y_{i}^{2}}$ and azimuth $\theta_{i}=\mathrm{atan}2\left(y_{i},x_{i}\right)$ are calculated. Each zone $Z_{m}$ is further subdivided radially into $N_{r,m}$ rings and circumferentially into $N_{s,m}$ sectors, forming grid cells $C_{m,(j,k)}$. The index for assigning point $p_{i}$ to a grid cell is determined by:

(3) $$j=\left\lfloor \frac{r_{i}-L_{m}^{\min}}{L_{m}^{\max}-L_{m}^{\min}}\cdot N_{r,m}\right\rfloor +1,\;k=\left\lfloor \frac{\theta_{i}}{2\pi /N_{s,m}}\right\rfloor +1$$

where *j* and *k* correspond to the indices of the radial ring and circumferential sector, respectively, and ⌊·⌋ denotes the floor operation. This non-uniform partitioning ensures high resolution in the near-field region while matching the sparse density of the far-field point cloud.

After grid partitioning, the system iterates through all non-empty grid cells and executes the MR-GPF algorithm. First, points within the cell are sorted in ascending order of height *z*, and the lowest $N_{\mathrm{ign},m}$ points are forcibly eliminated to remove the interference of subterranean reflection noise on the local plane fitting. Subsequently, the lowest $N_{\mathrm{lpr}}$ points are extracted from the remaining point cloud to calculate their average height ${\bar{z}}_{\mathrm{lpr}}$. Based on this height mean, the initial seed point set $S_{\mathrm{seed}}$ is defined as:

(4) $$S_{\mathrm{seed}}=\left\{p_{i}\in C_{m,(j,k)}|z_{i}<{\bar{z}}_{\mathrm{lpr}}+h_{\mathrm{seed}}\right\}$$

where $h_{\mathrm{seed}}$ is the seed point height margin. After extracting the initial seed points, Principal Component Analysis (PCA) is applied to solve for the unit normal vector n and the intercept *d* of the initial plane. To improve fitting robustness, the algorithm dynamically updates the inlier set based on the distance threshold $\tau_{d}$ and performs multiple iterations of fitting until convergence.

Upon convergence of the plane fitting, the final plane is translated upward along the normal vector direction by $\Delta h_{\mathrm{off}}$ to form an inclusive judgment baseline. The directed distance ${\mathrm{dist}}_{\mathrm{up}}$ from any point $p_{i}$ in the grid to this translated plane is calculated as follows:

(5) $${\mathrm{dist}}_{\mathrm{up}}=n\cdot p_{i}+\left(d-\Delta h_{\mathrm{off}}\right)$$

Primarily, valid ground points must be located below this translated plane, meaning the directed distance ${\mathrm{dist}}_{\mathrm{up}}<0$. Building upon this, to ensure the physical rationality of the segmentation results, these preliminarily filtered points must simultaneously satisfy two additional geometric constraints:

1. **Normal Uprightness:** The Z-axis component of the fitted plane is required to satisfy:
  (6) $$n_{z}>\gamma_{\mathrm{upright}}$$
  where $\gamma_{\mathrm{upright}}$ is a threshold close to 1, corresponding to an angle between the normal and the Z-axis less than $\arccos(\gamma_{\mathrm{upright}})$. However, this value is not set excessively close to 1 to better adapt to the undulating roads in unloading zones. Simultaneously, this geometric constraint effectively prevents the vertical chassis of large mining trucks from being erroneously misclassified as steep slopes.
2. **Local Flatness:** The standard deviation $\sigma_{\mathrm{dist}}$ of the distances from the ground points within the grid to the translated plane is calculated, and it is required to be less than the corresponding flatness threshold $\gamma_{\mathrm{flat},m}$ for that annular zone. It is worth emphasizing that this threshold is adaptively amplified as the radial distance of zone *m* increases. This zone-dependent dynamic design helps accommodate the measurement errors caused by the degradation of sensor spatial resolution at long distances, fully accommodating the natural undulations of unpaved mining roads over large spatial scales.

#### 3.4.2. Algorithm Formalization

The aforementioned unstructured terrain segmentation process based on physical geometric constraints is summarized in Algorithm 2.
**Algorithm 2** PatchWork-Mine Ground Segmentation**Require:** Fused point cloud $P_{fused}$; Thresholds $N_{val}$, $N_{\mathrm{ign},m}$, $N_{\mathrm{lpr}}$, $h_{\mathrm{seed}}$, $\tau_{d}$, $\Delta h_{\mathrm{off}}$, $\gamma_{\mathrm{upright}}$, $\gamma_{\mathrm{flat},m}$;CZM parameters $L_{m}^{\min},L_{m}^{\max},N_{r,m},N_{s,m}$.**Ensure:** Ground point cloud $P_{ground}$, Non-ground point cloud $P_{nonground}$.  1:$P_{valid}\leftarrow \mathrm{FilterNaNAndInf}\left(P_{fused}\right)$;  2:**if** $|P_{valid}|<N_{val}$ **then**  3:    **return** ∅, $P_{valid}$    *// Return early if valid points are insufficient*  4:**end if**  5:$P_{ground}\leftarrow \emptyset$;  6:$G\leftarrow \mathrm{CZM}\_\mathrm{Partition}(P_{valid},L_{m}^{\min},L_{m}^{\max},N_{r,m},N_{s,m})$;  7:**for** each non-empty grid cell $C_{m,(j,k)}\in G$ **do**  8:    $C_{clean}\leftarrow \mathrm{SortAndRemoveLowest}\left(C_{m,(j,k)},N_{\mathrm{ign},m}\right)$;  9:    ${\bar{z}}_{\mathrm{lpr}}\leftarrow \mathrm{CalculateLPRMeanHeight}\left(C_{clean},N_{\mathrm{lpr}}\right)$;10:    $S_{\mathrm{seed}}\leftarrow \left\{p_{i}\in C_{clean}|z_{i}<{\bar{z}}_{\mathrm{lpr}}+h_{\mathrm{seed}}\right\}$;11:    **if** $|S_{\mathrm{seed}}|<3$ **then**12:        **continue**;    *// Skip invalid PCA fitting*13:    **end if**14:    $n,d\leftarrow \mathrm{IterativePCAFit}(S_{\mathrm{seed}},\tau_{d})$;15:    $P_{temp}\leftarrow \left\{p_{i}\in C_{m,(j,k)}|n\cdot p_{i}+\left(d-\Delta h_{\mathrm{off}}\right)<0\right\}$;16:    **if** $|P_{temp}|>0$ **then**17:        $\sigma_{\mathrm{dist}}\leftarrow \mathrm{StandardDeviation}\left(\left\{n\cdot p+\left(d-\Delta h_{\mathrm{off}}\right)|p\in P_{temp}\right\}\right)$;18:        **if** $n_{z}>\gamma_{\mathrm{upright}}$ **and** $\sigma_{\mathrm{dist}}<\gamma_{\mathrm{flat},m}$ **then**19:            $P_{ground}\leftarrow P_{ground}\cup P_{temp}$;20:        **end if**21:    **end if**22:**end for**23:$P_{nonground}\leftarrow P_{valid}\setminus P_{ground}$;    *// The complement set represents non-ground points*24:**return** $P_{ground},P_{nonground}$;

### 3.5. Target Reconstruction and Quantitative Evaluation Framework

To facilitate the transition from low-level fused point clouds to high-level semantic understanding, a target reconstruction and geometric feature extraction framework is established in this section. This process provides the necessary prerequisites for calculating subsequent geometric evaluation metrics.

#### 3.5.1. Instance Reconstruction

First, the aforementioned PatchWork-Mine algorithm is employed for ground segmentation in unstructured mining terrain, effectively separating traversable areas from non-ground candidate obstacles. Second, the **DBSCAN** clustering algorithm is applied to the non-ground point cloud to group it into independent obstacle instances. Finally, for each extracted obstacle instance, the system generates a 3D Convex Hull to approximately represent its reconstructed spatial envelope and geometric boundaries. Based on Delaunay triangulation, the total volume *V* of the instance can be calculated by summing the volumes of the constituent tetrahedra of this convex hull.

#### 3.5.2. Quantitative Evaluation Metrics

To quantify the perception fidelity and multi-source fusion performance under extreme occlusion conditions, the following evaluation metrics are defined:

**Volume Recovery Rate (VRR)**

This proxy metric is introduced to quantify envelope-level spatial evidence for occluded obstacles. The calculation formula is:

(7) $$VRR=\frac{V_{fused}}{V_{\mathrm{ref}}}\times 100\%$$

where $V_{fused}$ denotes the convex-hull volume reconstructed from the fused point cloud, and $V_{\mathrm{ref}}$ denotes the reference bounding-envelope volume of the corresponding target. For irregular objects such as haul trucks, $V_{\mathrm{ref}}$ is defined as a conservative normalization reference derived from the external dimensions of the target. The reported VRR therefore represents an envelope-level proxy ratio of the fused point cloud, rather than a direct measure of complete object reconstruction accuracy. In this study, VRR is adopted as the main envelope-level indicator for NLOS spatial recovery under the tested mining conditions. It quantifies the recovered spatial evidence relative to the reference target envelope when LiDAR observations are incomplete or absent. When the VRR fluctuates around 100%, it indicates that the recovered point cloud envelope is close to the predefined reference envelope, and the point cloud exhibits neither significant absence nor over-inflation. If the VRR is significantly less than 100%, it implies incomplete spatial evidence in the reconstruction, mostly caused by local spatial holes or data sparsity due to severe occlusion. Conversely, if the VRR is significantly greater than 100%, it indicates an over-inflation of the reconstructed envelope, typically caused by unremoved clutter noise or multipath ghost points. Thus, under complete occlusion, a low but nonzero VRR indicates that the fused point cloud retains spatial evidence in the hidden-target region.

The relationship between VRR and commonly used perception metrics is summarized in Table 2. For the fully occluded NLOS scenes considered here, VRR evaluates recovered envelope-level spatial evidence without requiring complete object boxes, target centers, or voxel-level ground truth.

Accordingly, the subsequent experiments use VRR as a proxy indicator to compare envelope-level spatial recovery among LiDAR-only, radar-only, and fused point clouds in NLOS mining scenes.

**Chamfer Distance (CD) and Hausdorff Distance (HD):** CD and HD are employed to evaluate the geometric consistency between the fused point cloud and the LiDAR reference in observable regions, measuring the overall spatial similarity and the maximum local spatial deviation, respectively [27].

**Root Mean Square Error (RMSE) for cross-modal proximity and overlap-region consistency:** Nearest-neighbor RMSE is used in two ways in this study. Over all radar points, it reports scene-level cross-modal proximity after coordinate transformation, which is affected by non-overlapping NLOS radar observations. Within LiDAR-supported overlap regions, it reports calibration consistency for jointly observable points. In the MATLAB implementation, a KD-tree is constructed from the LiDAR point cloud transformed into the same coordinate frame, and each radar point is associated with its nearest LiDAR neighbor. The RMSE is calculated as

$$\mathrm{RMSE}=\sqrt{\frac{1}{N}\sum_{i=1}^{N}{∥p_{i}^{R}-{\mathrm{NN}}_{L}\left(p_{i}^{R}\right)∥}_{2}^{2}},$$

where $p_{i}^{R}$ is the *i*-th transformed radar point, ${\mathrm{NN}}_{L}\left(p_{i}^{R}\right)$ denotes its nearest LiDAR point, and *N* is the number of evaluated radar points. Since both point clouds are expressed in the same three-dimensional metric coordinate system, RMSE is reported in meters. The interpretation of RMSE therefore depends on whether it is computed over all radar points or only over the jointly observable overlap-region subset.

## 4. Experiments and Analysis

### 4.1. Experimental Platform and Sensor Configuration

The experiments were conducted in the real operating environment of the open-pit coal mine. The data acquisition platform is a drive-by-wire modified truck. A 1550 nm Innovusion Falcon Prime hybrid solid-state LiDAR is deployed on the roof of the vehicle to acquire 3D spatial point clouds of the environment. Simultaneously, to minimize signal distortion and multipath interference caused by the transmission and reception of electromagnetic waves interacting with the vehicle’s large metallic structures, an AMDR405T2 4D mmWave radar is mounted at the front of the vehicle, close to the bumper. The detailed hardware specifications are summarized in Table 3.

All module runtimes reported in this study were measured in MATLAB R2024b (MathWorks, Natick, MA, USA) on a Windows 11 workstation equipped with an AMD64 processor, 31.9 GB RAM, and an NVIDIA GeForce RTX 5070 Ti Laptop GPU. These results report the algorithmic processing time of the current MATLAB implementation and provide a benchmark for subsequent embedded deployment.

**Figure 2:**
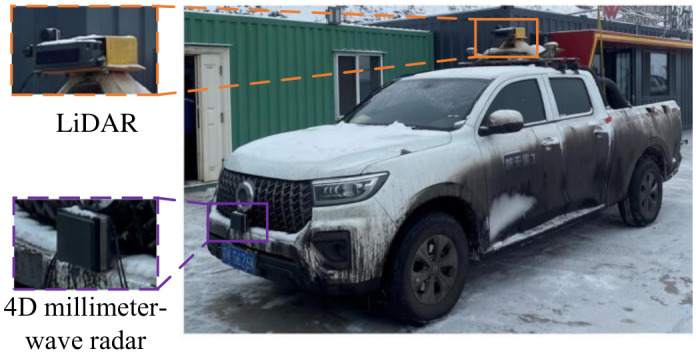
Vehicle Sensor Configuration for Data Acquisition.

**Figure 3:**
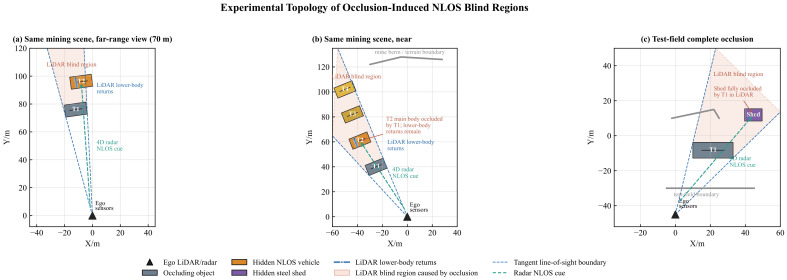
Experimental topology of occlusion-induced NLOS blind regions. The mining experiments include the far-range view at 70 m and the near-range view at 35 m in the same unloading-zone scene, while the test-field experiment uses a foreground haul truck to occlude the steel shed.

The sensor mounting configuration of the experimental vehicle is shown in Figure 2. Figure 3 summarizes the occlusion topology of the three representative experimental conditions used in this study. The far-range and near-range mining experiments correspond to the same unloading-zone scene observed at approximately 70 m and 35 m, respectively. In these two mining cases, the foreground truck blocks the main body of the rear target from direct LiDAR observation, while a small number of low-height LiDAR returns remain near the lower vehicle body. The test-field experiment uses a foreground haul truck to fully occlude a steel shed in the LiDAR line of sight. These layouts provide representative NLOS conditions for evaluating blind-region radar cue injection and envelope-level spatial recovery.

### 4.2. Experimental Scenarios and Baseline Volume Calibration

To verify the occlusion compensation capability of the fusion algorithm under various operating conditions, three sets of occlusion perception experiments are designed. Considering the significant size differences among the models acting as target obstacles in different experimental scenarios, to ensure the objectivity and comparability of the Volume Recovery Rate (VRR) metric, this section independently defines the reference bounding-envelope volume ($V_{\mathrm{ref}}$) used for normalization:

- **Real mining scenario experiments:** The target obstacle in these experiments is a Komatsu 930E large mining haul truck. With a length, width, and height of 15.6 m, 8.7 m, and 7.4 m, respectively, its reference bounding-envelope volume $V_{\mathrm{ref}}$ is approximately 1004.3 m^3^. This value is used as a conservative geometric envelope for VRR normalization.
- **Test track simulation scenario experiments:** The target obstacles in this set of experiments include a “Zaishan”(CarMo) new energy autonomous mining truck and a steel shed. The length, width, and height of the CarMo truck are 8.9 m, 3.5 m, and 3.4 m, respectively, yielding a reference bounding-envelope volume $V_{\mathrm{ref}}$ of approximately 105.9 m^3^. Based on on-site measurements, the reference envelope volume $V_{\mathrm{ref}}$ of the steel shed is approximately 48 m^3^.

By establishing this $V_{\mathrm{ref}}$ baseline tied to the external dimensions of specific targets, subsequent experiments can consistently quantify the envelope-level spatial recovery capability of the BSCF algorithm when dealing with mining equipment of varying scales. Accordingly, the reported VRR values describe normalized recovery ratios with respect to the reference obstacle envelope.

### 4.3. System Pipeline Performance Evaluation

To clearly demonstrate the execution process and intermediate results of the proposed algorithm pipeline, this section selects a typical single frame of data collected from the open-pit coal mine for step-by-step analysis, thereby verifying the qualitative processing effects of each module. This representative frame corresponds to mine1, namely the far-range mining scene with an approximately 70 m target distance. The camera view, intermediate point-cloud figures, clustering result, and obstacle table in this section are interpreted for the same single-frame scene so that the processing stages can be compared consistently.

#### 4.3.1. Preprocessing and Spatial Alignment

**Figure 4:**
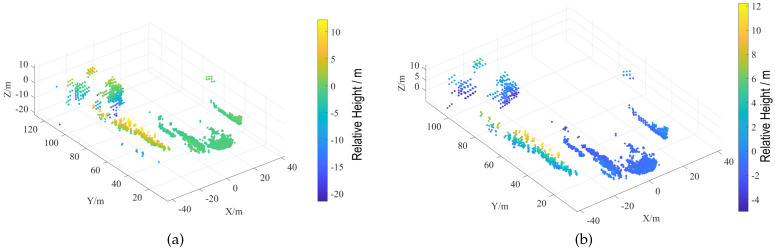
4D mmWave radar point cloud preprocessing in the far-range mining scene (70 m): (**a**) raw radar point cloud before ground-plane filtering; (**b**) preprocessed radar point cloud after low-altitude ghost suppression. Coordinates are in meters.

As shown in Figure 4, the raw 4D mmWave radar point cloud contains a massive amount of multipath reflection clutter originating from the ground, with relative height values reaching as low as −20 m, which severely deviates from the true physical ground. Through the robust plane fitting algorithm applied in the preprocessing stage, such subterranean ghost points are directly filtered out. The minimum relative height of the valid point cloud converges to −4 m, physically eliminating the interference of invalid data on subsequent algorithms.

**Figure 5:**
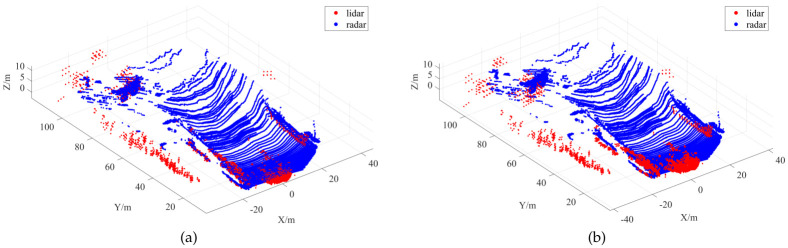
LiDAR–radar spatial synchronization under occlusion conditions: (**a**) before calibration; (**b**) after calibration. Red and blue points denote LiDAR and 4D mmWave radar points, respectively. Coordinates are in meters.

Regarding multi-sensor coordinate transformation and synchronization, the alignment comparison in Figure 5 illustrates that before spatial calibration, there is an obvious spatial misalignment between the LiDAR point cloud (red) and the mmWave radar point cloud (blue). Following spatiotemporal synchronization calibration, the heterogeneous point clouds show improved scene-level cross-modal proximity in partially visible regions. Quantitative data indicate that the all-point nearest-neighbor Root Mean Square Error (RMSE) between the two sets before calibration was 6.25 m, which decreases to 5.22 m after calibration, representing a scene-level proximity improvement of approximately 16.5%. This all-point RMSE is calculated from nearest-neighbor distances between the transformed radar points and the LiDAR point cloud in the same coordinate frame, and is used here as a cluster-level proximity indicator for the real mining scene shown in Figure 5, rather than as a pure calibration-error metric. To distinguish calibration consistency from non-overlapping NLOS observations, an overlap-region diagnostic was further calculated using radar points whose nearest-neighbor distance to LiDAR after calibration was no larger than 0.3 m. In the three mining scenes, this LiDAR-supported subset yielded post-calibration RMSE values of 0.175–0.180 m, indicating good coordinate consistency in jointly observable regions. In contrast, the all-point RMSE remains at the meter level because it is affected by LiDAR-invisible NLOS radar returns and non-overlapping radar observations. Therefore, the global RMSE is interpreted as a cluster-level proximity indicator rather than a pure calibration-error measure, and the calibrated point clouds are further processed by radar spatial consistency filtering and LiDAR blind-zone verification before blind-region injection.

#### 4.3.2. BSCF Blind-Spot Complementary Fusion

**Figure 6:**
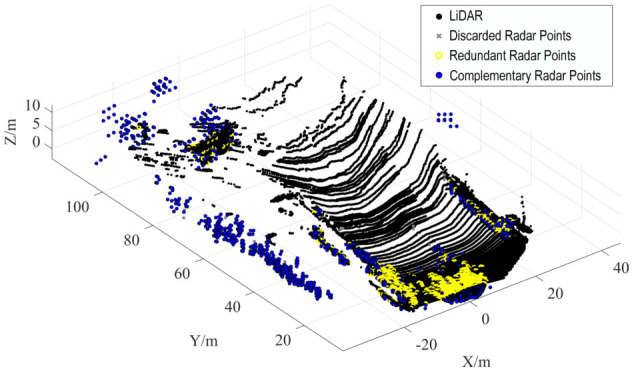
Point-cloud filtering based on BSCF in the far-range mining scene (70 m). Black points denote downsampled LiDAR points, cross marks denote isolated radar noise, yellow points denote LiDAR-visible redundant radar points, and blue points denote retained blind-region complementary radar points. Coordinates are in meters.

Figure 6 demonstrates the hierarchical filtering process of the radar point cloud: black points represent the downsampled LiDAR point cloud, where the number of points drops from 40,227 to 32,033, optimizing computational efficiency while retaining scene features; cross marks indicate isolated noise points that failed the spatial consistency verification; yellow points denote redundant radar points that satisfy spatial consistency but fall within the LiDAR’s field of view, which are discarded to prevent the degradation of the system’s overall depiction accuracy; blue points are the final retained valid complementary radar points.

The fusion statistics reveal that out of the initial 7379 radar points, 7362 spatially consistent points are retained. Furthermore, through blind-spot determination, 4126 blind-spot complementary points are selected in this representative frame. The final fused point cloud totals 36,159 points, with a BSCF filtering time of 30.8 ms in this single-frame example. The spatial coverage under occlusion conditions is improved by 12.9% compared to using LiDAR alone. These results indicate that BSCF can selectively supplement the occlusion blind spots of LiDAR while curbing the interference of redundant radar points on fusion accuracy.

#### 4.3.3. PatchWork-Mine Ground Segmentation

The PatchWork-Mine algorithm is executed for traversable area segmentation, with a single-frame processing time of 86.34 ms. The segmentation results yield a ground point set accounting for 71.5% and a non-ground point set accounting for 22.6%, separating the primary ground structures in the complex environment.

**Figure 7:**
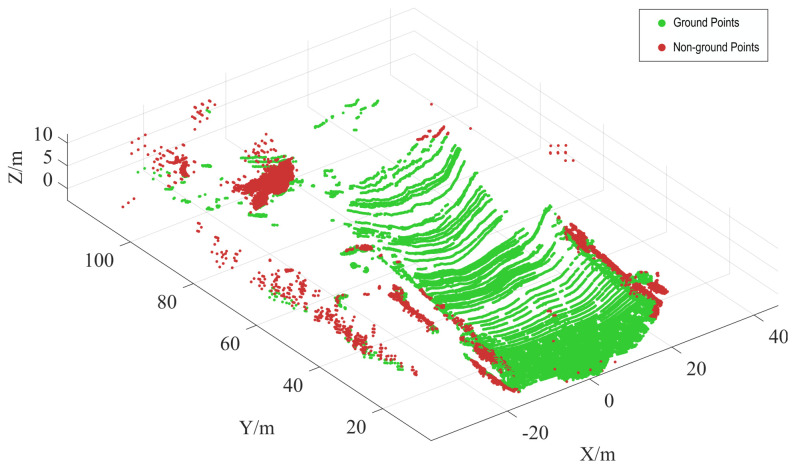
PatchWork-Mine ground segmentation result in the far-range mining scene (70 m). Coordinates are in meters.

The results in Figure 7 show that the extracted ground point cloud coherently adheres to the actual undulating terrain, without obvious over-segmentation at irregular boundaries such as retaining walls and slopes. Simultaneously, based on the built-in parameter filtering and translation constraint mechanisms, the ground fitting in noise-dense areas remains robust. This prevents the vertical features of large obstacles, such as mining trucks, from being misclassified as slopes, providing a high-purity non-ground point cloud for subsequent clustering.

Because this study targets long-range, uneven, and slope-dominated unloading roads in open-pit mines, PatchWork-Mine is treated as a mining-specific adaptation of Patchwork-family ground segmentation rather than as a general replacement for Patchwork or Patchwork++. The main design differences are summarized in Table 4. This design-level comparison highlights the mining-specific adaptations, including zone ranges, plane thresholds, and low-point handling for steep and uneven unloading terrain.

#### 4.3.4. Obstacle Clustering and Feature Extraction

**Figure 8:**
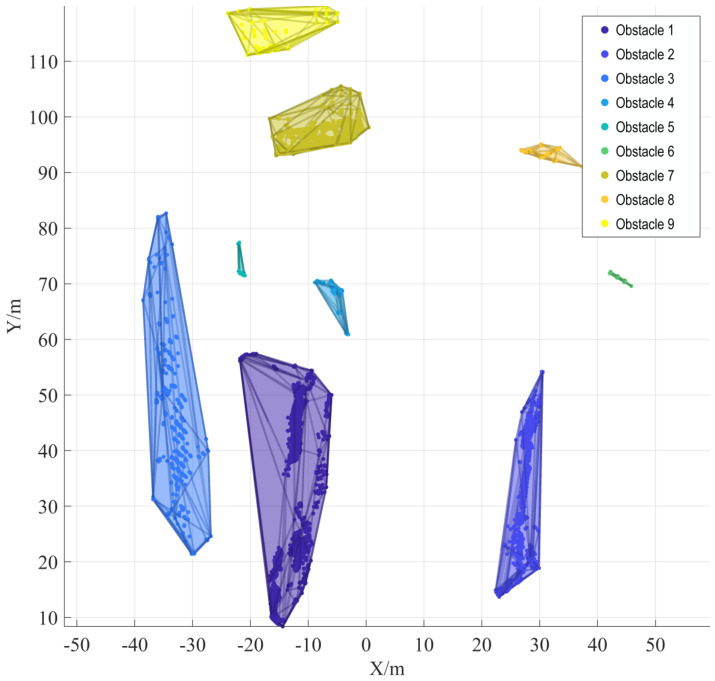
Obstacle clustering and convex-hull extraction result in the far-range mining scene (70 m). The obstacle IDs correspond to the instance list in Table 5. Coordinates are in meters.

Based on the DBSCAN algorithm applied to the non-ground point cloud, the targets in the scene are divided into 9 main obstacle clusters, covering the foreground occluding truck, the rear occluded truck, stepped road benches, roadside soil slopes, road boundaries, and local road-surface undulations. Furthermore, a convex hull generation algorithm is applied to each obstacle cluster to extract the 3D boundary polyhedrons (Figure 8), intuitively presenting the spatial contours and relative positions of the obstacles. The obstacle numbers visualized in Figure 8 correspond to the instance IDs and manually interpreted categories listed in Table 5.

Table 5 details the geometric feature information of these 9 obstacle instances. To improve interpretability, a manual category column is added based on visual inspection of the scene and the relative position of each cluster. These manually interpreted category names are used only to explain the scene content in the evaluation figures and do not indicate that the clustering algorithm performs automatic semantic classification. The data reveals a positive correlation between the point cloud quantity and the target’s reference envelope size, though not strictly linear. This objectively reflects the combined influence of target materials, surface reflection characteristics, and sensor viewing angles on the perception density.

### 4.4. Real-World Mining Unloading Zone Validation

**Figure 9:**
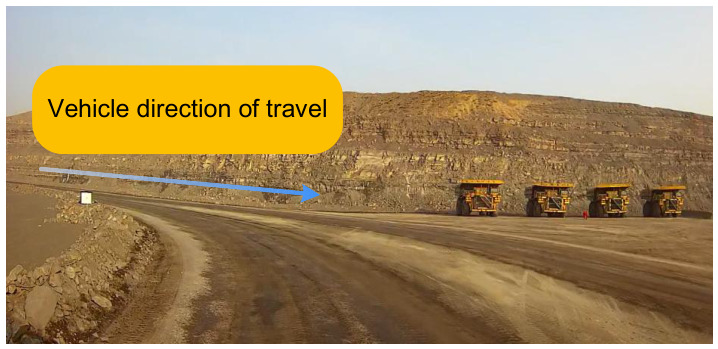
Real-world mining unloading-zone experimental scene used for the far-range and near-range occlusion tests.

This set of experiments focuses on the unloading zone of the open-pit mine. This area frequently features multiple mining trucks parked side-by-side, presenting severe mutual occlusion. The following far-range and near-range experiments use the camera views and corresponding point-cloud results jointly: the camera images indicate the physical occlusion relationship, while the point-cloud subfigures show how the same scene is perceived by fused, LiDAR-only, and radar-only inputs. The real-world mining unloading-zone scene used for these two occlusion tests is shown in Figure 9.

#### 4.4.1. Far-Range (70 m) Performance

**Figure 10:**
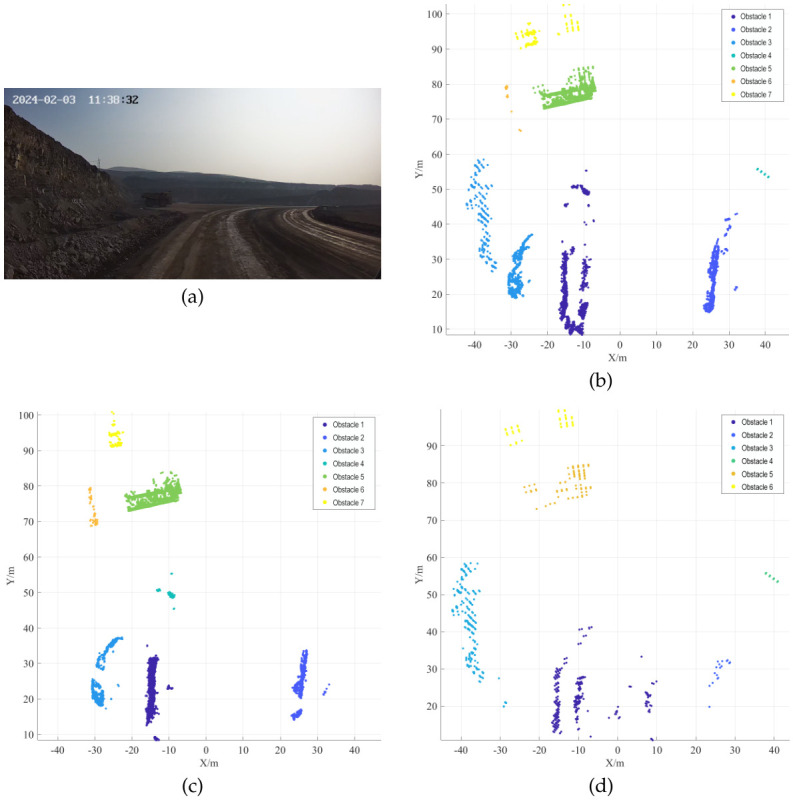
Visual comparison of the far-range (70 m) mining experiment: (**a**) camera view of the occlusion scene; (**b**) fused point-cloud clustering result; (**c**) LiDAR-only point-cloud clustering result; (**d**) 4D mmWave radar-only point-cloud clustering result. Point-cloud coordinates are in meters.

At a detection distance of 70 m, the density of the LiDAR point cloud attenuates significantly with distance, resulting in a severe lack of observation data for the occluded trucks in the rear. As shown in Figure 10, the volume recovery rates of the two unmanned mining trucks from near to far are denoted as VRR1 and VRR2, respectively. In this scene, VRR1 corresponds to the front visible truck, whereas VRR2 corresponds to the rear truck that is more strongly occluded in the camera view and point-cloud results. The data in Table 6 indicate that for the first truck (Obstacle 5), the VRR1 of the fused point cloud is 72.7%, which is higher than that of the LiDAR (53.8%) and the raw mmWave radar (23.8%). For the second, more distant and severely occluded truck (Obstacle 7), the LiDAR’s VRR2 drops to 5.0%, making it difficult to recover an effective reference-envelope cue; the raw mmWave radar’s VRR2 is 18.2%, showing limited geometric recovery capability. In contrast, the VRR2 of the fused point cloud under these extreme conditions is maintained at 59.3%. Using VRR as the main envelope-level recovery metric, the results show that BSCF recovers more blind-region spatial evidence than LiDAR-only or radar-only perception in the far-range occlusion scene.

In this and subsequent quantitative result tables, bold values indicate the proposed BSCF results or the most desirable diagnostic values for the corresponding metric.

#### 4.4.2. Near-Range (35 m) Performance

**Figure 11:**
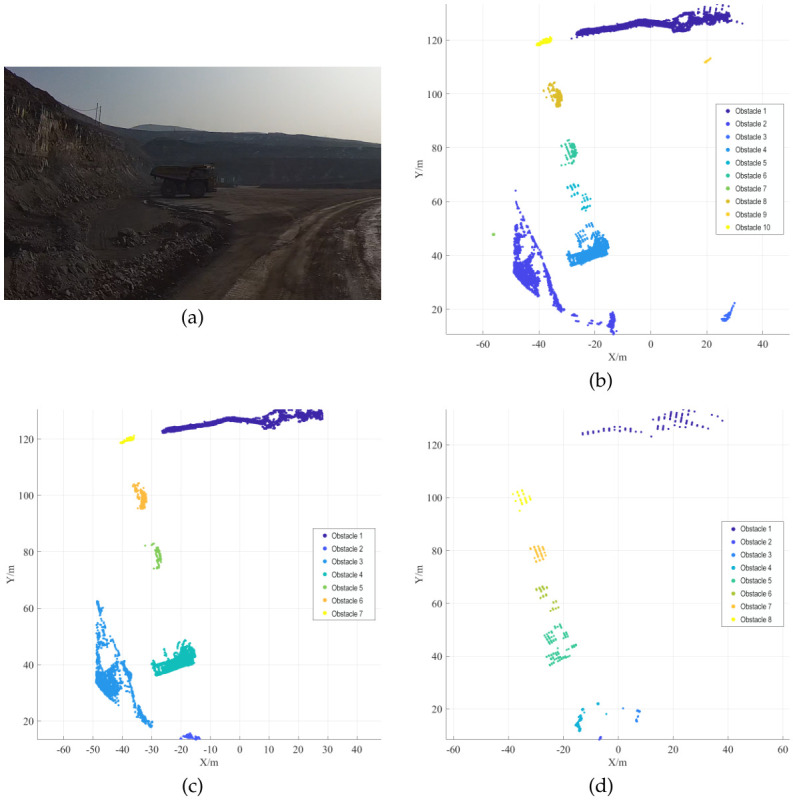
Visual comparison of the near-range (35 m) mining experiment: (**a**) camera view of the multiple-truck occlusion scene; (**b**) fused point-cloud clustering result; (**c**) LiDAR-only point-cloud clustering result; (**d**) 4D mmWave radar-only point-cloud clustering result. Point-cloud coordinates are in meters.

In the 35 m near-field scenario, the LiDAR exhibits dense spatial depiction capability for unoccluded targets; however, limited by its optical line-of-sight characteristics, it yields zero detection for the second truck, which is completely occluded by the front vehicle. This set of experiments denotes the volume recovery rates of the four unmanned mining trucks from near to far as VRR1, VRR2, VRR3, and VRR4, respectively. The numbering follows the relative front-to-back order in the camera view and the corresponding point-cloud clusters, allowing the VRR values in Table 7 to be read together with Figure 11. For the nearest first truck, the fused point cloud achieves a VRR1 of 90.2%, outperforming the LiDAR (60.1%) and the mmWave radar (36.7%). For the second truck, the LiDAR VRR2 data is missing (physical missed detection), and the raw mmWave radar’s VRR2 is 10.2%; the fused point cloud provides an existence-level risk cue for the target and recovers 15.6% of its reference envelope volume. For the third and fourth trucks located further away under multiple occlusions, the VRR3 (17.8%) and VRR4 (18.7%) of the fused point cloud consistently outperform those of the single sensors. The above data indicate that in near-field operational scenarios involving multiple occlusions, the fusion perception scheme can improve the reference-envelope recovery ratio and hidden-target risk-cue coverage within blind spots through heterogeneous information complementarity. In the completely occluded second-truck case, the nonzero VRR of the fused point cloud indicates that BSCF retains weak but useful spatial evidence in the LiDAR blind region.

#### 4.4.3. Geometric Consistency Validation in Mining Scenes

Furthermore, Table 8 records the geometric consistency metrics under these two real-world scenarios. In the table, *F*, *R*, and *L* represent the Fused point cloud, the raw mmWave Radar point cloud, and the reference high-precision LiDAR point cloud, respectively. The data show that the Chamfer distance CD(F,L) and Hausdorff distance HD(F,L) of the fused point cloud in both scenarios are lower than the CD(R,L) and HD(R,L) of the raw radar. For scenes containing fully hidden targets, these CD and HD values are used to evaluate visible-region consistency and multipath suppression in the observable parts of the scene. Notably, the HD value, which is extremely sensitive to outlier noise, decreases significantly. This intuitively reflects that the BSCF framework effectively suppresses the maximum local spatial deviation caused by multipath ghosting while retaining the radar’s penetrative information.

### 4.5. Test Field Simulation Under Extreme Occlusion

**Figure 12:**
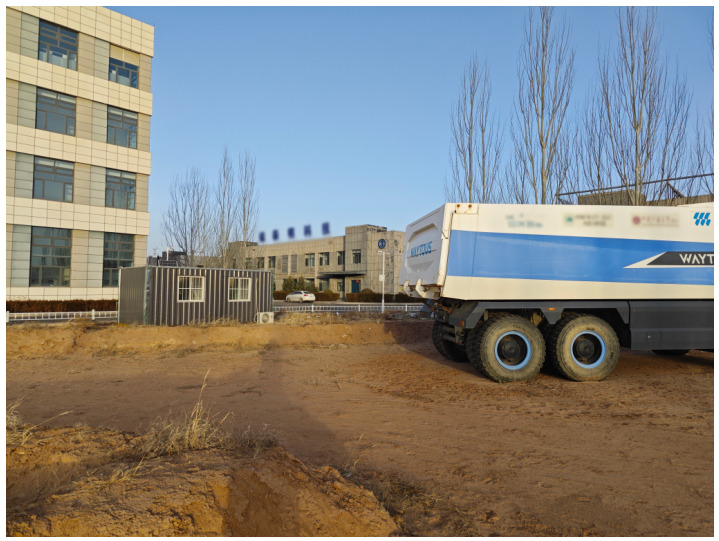
Test-field extreme occlusion environment. The steel shed is fully occluded by the foreground haul truck in the camera view. Any non-English site signage visible in the image denotes site/operator information and is not an experimental variable.

**Figure 13:**
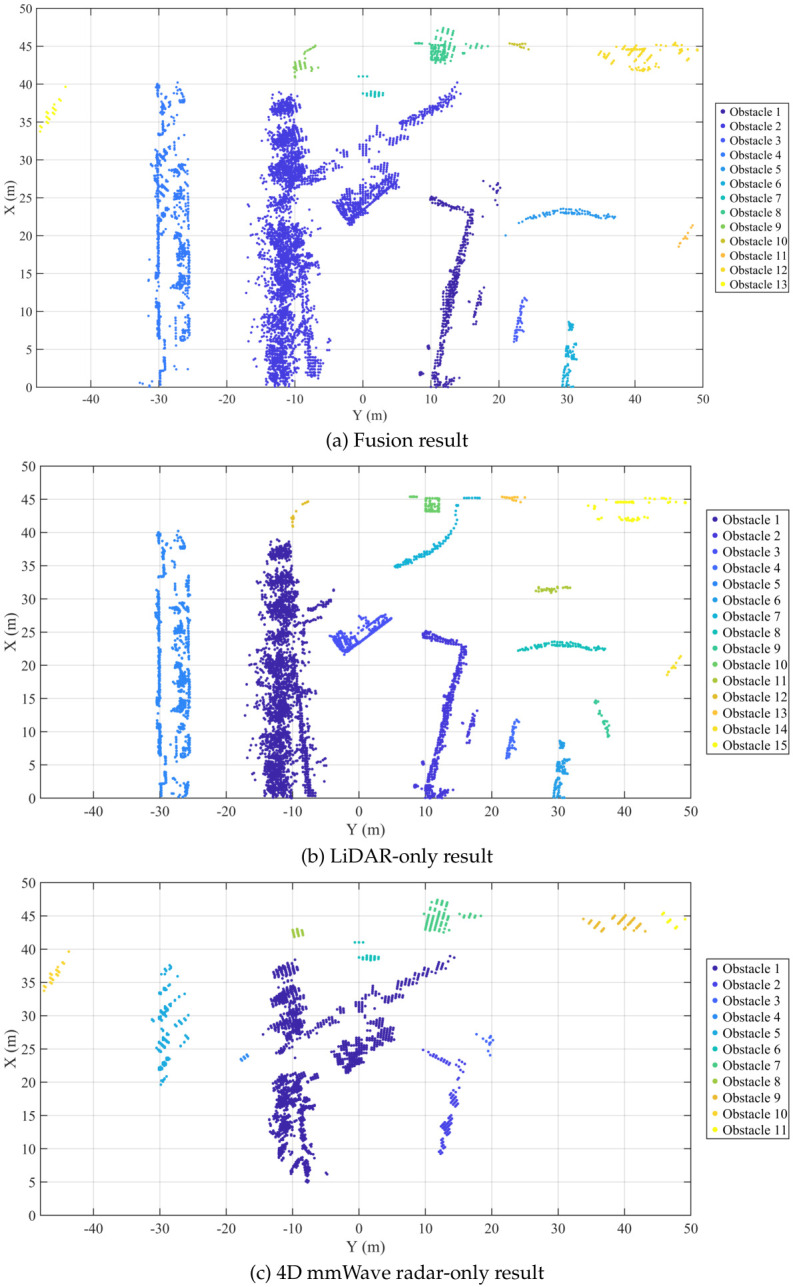
Point-cloud comparison of the test-field extreme occlusion experiment using enlarged top-view renderings rotated by 90 degrees: (**a**) fused point cloud; (**b**) LiDAR-only point cloud; (**c**) 4D mmWave radar-only point cloud. The horizontal and vertical axes correspond to *Y* and *X*, respectively, and coordinates are in meters.

To test the boundary capabilities, a steel shed was completely occluded by a haul truck. While LiDAR failed to detect the shed, BSCF produced a weak but nonzero hidden-target cue through the penetrative capability of 4D radar. As shown in Table 9, the test-field VRR values report foreground-truck and hidden-shed envelope recovery, while Table 10 reports the corresponding geometric consistency metrics. In this controlled test-field scene, VRR1 refers to the foreground haul truck, and VRR2 refers to the steel shed hidden behind it. The camera image in Figure 12 and the point-cloud comparison in Figure 13 should therefore be interpreted as the same front-truck/hidden-shed occlusion layout.

### 4.6. Ablation Study

After evaluating the far-range mining scene, the near-range mining scene, and the test-field extreme occlusion scene, an ablation study is conducted on these three representative scenarios to examine the contribution of the main BSCF filtering components. The preceding experiments use VRR, CD, and HD as performance-level indicators, while this ablation reports radar-source diagnostics to show how each component affects blind-region radar injection, visible-region leakage, and isolated radar noise. The three ablation scenarios correspond to the same experimental categories analyzed above: far-range mining occlusion, near-range multi-truck occlusion, and test-field truck-shed occlusion.

For the BSCF filtering ablation, injected radar points denote radar-source points retained after each fusion variant. Visible leakage points denote radar-source points whose nearest LiDAR distance is smaller than the blind-region threshold, indicating redundant radar injection in LiDAR-visible regions. Isolated radar points denote radar-source points with fewer than the required spatial neighbors. Table 11 summarizes the point-count diagnostics for the three ablation scenarios before the ROI-level and radar-preprocessing ablations are reported.

**Figure 14:**
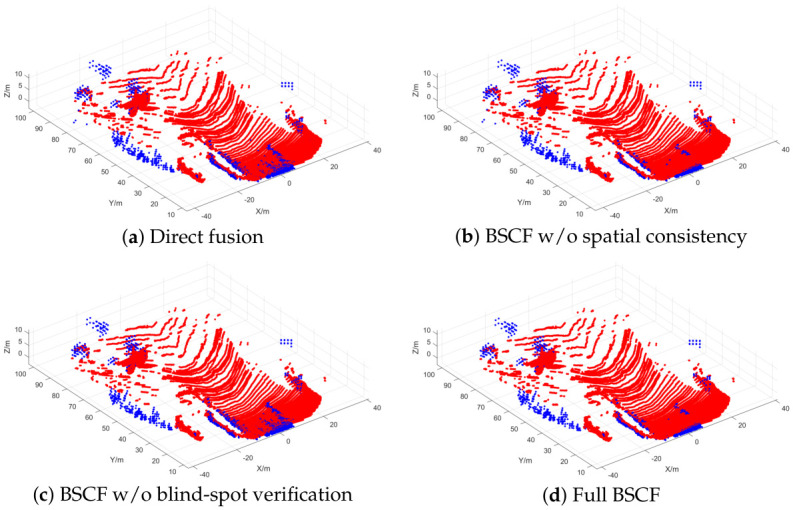
BSCF filtering ablation visualization in the far-range (70 m) mine scene. Red points denote LiDAR points, and blue points denote radar-source points retained by each variant. Coordinates are in meters.

**Figure 15:**
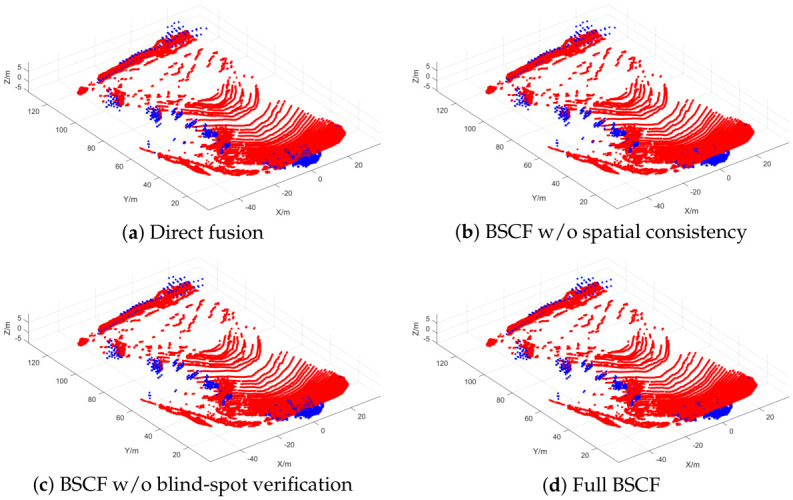
BSCF filtering ablation visualization in the near-range (35 m) mine scene. Red points denote LiDAR points, and blue points denote radar-source points retained by each variant. Coordinates are in meters.

**Figure 16:**
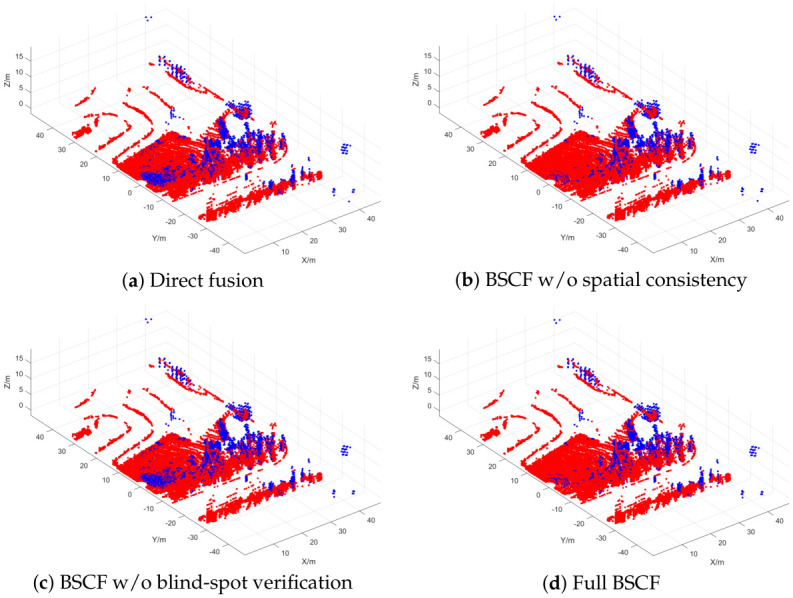
BSCF filtering ablation visualization in the test-field extreme occlusion scene. Red points denote LiDAR points, and blue points denote radar-source points retained by each variant. Coordinates are in meters.

Figure 14, Figure 15 and Figure 16 visualize the corresponding ablation results for the far-range mining, near-range mining, and test-field scenarios, respectively.

The ROIs used in Table 12 are manually associated using the known field layout, camera observations, the relative position of the occluding truck, and the expected hidden-target location. This table reports radar cue counts within manually associated haul-truck target ROIs for the mining sequences, rather than automatic semantic detection results.

A radar-side preprocessing ablation is also reported for the mine point-cloud sequences in which raw, filtered, and aligned radar stages are available. Low-altitude ghost points are defined as radar points with a relative height below −4 m, which mainly correspond to physically implausible below-ground multipath artifacts in the mining scene. The −4 m threshold is chosen according to the physical ground-plane constraint and the post-filtering ground-height range observed in Figure 4. The corresponding point-count results are summarized in Table 13.

The representative single-frame timings reported above, including the 30.8 ms BSCF filtering time and the 86.34 ms PatchWork-Mine segmentation time, correspond to the specific visualized examples in the pipeline analysis. Table 14 reports an averaged MATLAB benchmark over repeated runs on the mine1 frame; therefore, differences between the representative single-frame values and the averaged module-level values reflect frame content and benchmarking protocol rather than conflicting measurements.

## 5. Discussion

This section will discuss the theoretical advantages and engineering application values demonstrated by the BSCF framework in complex mining environments, as well as the objective limitations of the current algorithm.

### 5.1. Physical Interpretation of Multipath Suppression

Experimental results indicate that multipath interference is one of the primary factors affecting radar perception reliability in open-pit mining environments. The massive metallic equipment and irregular terrain in mines form complex multipath propagation routes, thereby generating numerous spurious targets beneath the ground surface, which further distort local geometric structures and increase the difficulty of obstacle identification. The BSCF framework proposed in this paper mitigates this issue through a two-stage strategy. First, the RANSAC-based preprocessing removes a large portion of isolated outliers and unstable radar reflections. Subsequently, the traversable-area model established by PatchWork-Mine demonstrates strong robustness against local geometric distortions. By combining radar reflection characteristics with terrain-aware geometric constraints, this method effectively suppresses multipath-induced artifacts while preserving useful obstacle information.

On the other hand, while providing penetration information, multipath effects also exert a specific impact on the geometric boundaries of targets. The volume expansion phenomenon observed in the test field scenario primarily originates from the strong electromagnetic multipath reflections generated between the truck’s massive metallic body and the steel shed behind it. From the perspective of risk asymmetry in autonomous driving perception systems, this spatial perception false positive caused by radar characteristics essentially introduces a conservative safety margin for the system in high-risk mining environments, prompting the downstream planning module to adopt safer evasive strategies. Compared to the potential safety risks caused by missed detections of hidden targets, the conservative perception results resulting from moderate spatial expansion are more acceptable from an engineering safety perspective. Therefore, the objective of the perception system is not to eliminate all multipath responses, but rather to suppress spurious multipath artifacts while preserving reflection information that may indicate the presence of potentially hidden targets. Accordingly, a VRR value exceeding 100% indicates envelope over-recovery or conservative risk coverage. The scenario-wise ablation results in Table 11 show that direct fusion introduces visible-region radar leakage in all three tested scenarios, whereas the complete BSCF keeps visible leakage at zero while retaining radar-source points in blind regions. This result indicates that blind-spot verification converts radar supplementation from simple point-cloud concatenation into selective blind-region cue injection. The ROI-based cue counts in Table 12 further show that the complete BSCF preserves hidden-target radar cues in the associated mining-scene ROIs while removing visible-region leakage. The radar-side ablation in Table 13 illustrates the role of preprocessing and spatial consistency filtering in suppressing below-ground ghost points and isolated radar returns before blind-region supplementation.

### 5.2. Geometric Constraints and Blind-Spot Completion Mechanism

In addition to multipath suppression, the BSCF framework effectively addresses the severe asymmetric visibility challenge. In mining scenarios, large mining trucks, excavators, or terrain structures may completely occlude the line-of-sight of the LiDAR, leading to perception blind spots. Traditional perception fusion frameworks typically rely on spatial alignment and feature concatenation between multi-source data. When complete physical occlusion causes LiDAR observation data to be zero, such fusion mechanisms relying on a common observation foundation often fail due to the loss of a reference baseline. Unlike fusion methods that primarily rely on cross-modal feature alignment, this paper introduces explicit geometric constraints based on spatial consistency filtering. By complementing the detection mechanisms of heterogeneous sensors, the system utilizes the penetration characteristics of the mmWave radar to directly acquire point cloud data within optically occluded areas, and effectively extracts it through explicit spatial consistency filtering, thereby inferring the existence of hidden obstacles. As demonstrated by the data from the near-range experiments in real mining scenarios and the extreme occlusion experiments in the test field, the results indicate that this method can recover partial spatial observation information of targets under occlusion conditions where the LiDAR completely fails, thereby potentially reducing missed-risk under the tested scenarios. This blind-spot completion strategy guided by geometric consistency demonstrates that explicit geometric constraints can effectively compensate for the observation deficiency of traditional fusion frameworks in completely occluded scenarios, enhancing perception robustness under extreme occlusion conditions.

Experimental results demonstrate that multipath suppression and explicit geometric constraints are two key factors in achieving occlusion perception in mines. The former improves the physical reliability of perception results, while the latter enhances the target existence inference capability under extreme occlusion conditions. The synergistic effect of both enables the BSCF framework to maintain high perception robustness in complex mining environments. However, how to further utilize high-order multipath information to achieve fine-grained reconstruction of hidden targets remains an important direction worthy of in-depth study in the future.

### 5.3. Limitations and Future Work

From the perspective of downstream autonomous haulage, low VRR values such as 15.6% or 5.2% serve as conservative risk cues for hidden-target existence. In practical operation, such cues are most suitable for risk-aware behaviors such as speed reduction, enlarged safety margins, active re-observation, or a switch to a more cautious planning mode in unloading areas.

The current validation focuses mainly on large mining trucks and a steel shed. Since radar reflectivity, multipath behavior, and point-cloud sparsity vary substantially across object categories, generalization to pedestrians, small vehicles, rock-pile boundaries, excavator components, and other mining equipment still requires additional category-level experiments.

Although the proposed method improves occlusion perception in the tested mining scenarios, the current prototype still has several limitations, which also indicate important directions for future research:

1. **Insufficient fine-grained geometric representation capability for hidden targets:** As shown in Table 7 and Table 9, for completely occluded targets, the system’s volume recovery rate is typically below 20%. Although this provides a conservative indication of target existence, the high sparsity of the penetrative radar point cloud makes it difficult for the current algorithm to perform precise 3D Bounding Box Regression or fine-grained semantic classification for hidden targets.
2. **The challenge of completely eliminating high-order multipath inflation:** The volume inflation (108.6%) observed in Table 9 indicates that relying solely on spatial distance-based geometric consistency filtering is still insufficient to completely strip away high-order multipath reflections generated by the coupling between large metallic equipment.
3. **Clustering efficiency for embedded deployment:** The runtime benchmark shows that the current end-to-end prototype is dominated by the DBSCAN clustering stage, which takes 403.55 ms for 10,605 non-ground points and accounts for approximately 72.2% of the total MATLAB processing time. Therefore, subsequent deployment should prioritize optimized neighborhood search, parallel clustering, and temporal object tracking while retaining the moderate runtimes of the BSCF and PatchWork-Mine modules.
4. **Validation under degraded visibility:** The current experiments were conducted under normal visibility conditions using data collected in a real operating open-pit mine in 2024. No dust-, rain-, fog-, or snow-specific experiment is included in the present validation because additional field acquisition under such conditions requires site access, production coordination, and safety approval, while natural degraded-weather events are difficult to schedule within a short validation period. Dust, rain, fog, snow, and airborne particles may change LiDAR point density, radar scattering behavior, and multipath characteristics in open-pit mines. Future work will therefore evaluate the proposed framework under controlled and field-measured degraded-visibility conditions before deployment in all-weather autonomous haulage, following prior LiDAR adverse-weather and filtering studies, including Sensors work on automotive LiDAR weather influence [28,29,30,31,32].

Future work will focus on transitioning from simple outlier removal to explicit multipath modeling. By integrating physics-based ray-tracing models with machine learning techniques, high-order multipath propagation mechanisms in complex environments can be estimated more accurately. Such an approach may enable multipath reflection points to be mapped back to their true reflection sources, thereby reducing multipath-induced spatial inflation while simultaneously exploiting hidden target information contained in multipath signals. Ultimately, this could further improve the geometric reconstruction capability of completely occluded targets. In parallel, future engineering work will replace the current MATLAB DBSCAN implementation with more efficient clustering strategies, such as voxel-indexed region growing, GPU-parallel neighborhood search, or incremental object tracking across frames, and will validate the complete latency on the target autonomous haulage controller.

## 6. Conclusions

This paper addresses the critical environmental perception challenges, such as extreme occlusion and high-order multipath interference, faced by unmanned mining trucks in the unloading zones of open-pit mines. A Blind-Spot Complementary Fusion (BSCF) framework integrating 3D LiDAR and 4D millimeter-wave (mmWave) radar is proposed. Based on real-world unloading zone scenarios and test field experiments at the Haerwusu Open-pit Coal Mine, the main conclusions are drawn as follows:

1. **Physical Constraints and Cluster-Level Spatial Synchronization:** The proposed RANSAC-based denoising mechanism suppresses high-order multipath ghosting caused by the coupling of metallic equipment and the ground. It raises the minimum relative height of valid radar points from −20 m to −4 m, improving the underlying data quality. Furthermore, the MNDT-based spatial synchronization algorithm reduces the all-point nearest-neighbor RMSE of the heterogeneous sensors from 6.25 m to 5.22 m, achieving an approximately 16.5% improvement in scene-level cross-modal proximity. In the jointly observable overlap-region subset, the post-calibration RMSE remains approximately 0.18 m, providing a calibration-consistency basis for the 0.3 m blind-region threshold and subsequent blind-region cue injection.
2. **Complex Occlusion Penetration and Existence Capture:** The BSCF framework effectively extracts the penetrative data of the mmWave radar in optically occluded areas through heterogeneous sensor information complementarity. Under the 70 m far-range observation, the fusion algorithm increases the volume recovery rate (VRR) of the target from 5.0% (single LiDAR) to 59.3%. In the 35 m near-range multiple occlusion and the test field extreme occlusion scenarios, the fusion algorithm retains sparse envelope-level radar evidence corresponding to 15.6% and 5.2% of the reference envelope volumes of targets located within the complete physical missed detection zone of the LiDAR. These low VRR values should be interpreted as existence-level hidden-target risk cues for downstream risk assessment, not as complete hidden-target reconstruction. This indicates that the system can provide existence-level hidden-obstacle warnings under physical occlusion.
3. **Spatial Error Constraint and Geometric Consistency:** Under complex occlusion conditions, the Chamfer distance CD(F,L) between the fused point cloud and the LiDAR reference point cloud in observable regions is only 1.62, and the Hausdorff distance HD(F,L) is 21.55 m. Both metrics are lower than the corresponding 27.82 and 27.51 m values of the raw mmWave radar. These metrics indicate visible-region consistency and multipath suppression in scenes where fully occluded objects have no direct LiDAR observations. The ablation study further confirms that BSCF suppresses visible-region radar leakage while preserving radar cue points in the target ROI.
4. **Unstructured Terrain Robustness and Runtime Characteristics:** The introduced PatchWork-Mine algorithm demonstrates robust ground segmentation capability on undulating real-world mining surfaces, providing a useful geometric baseline for subsequent instance extraction. In the MATLAB runtime benchmark, the BSCF filtering module takes 39.23 ms, PatchWork-Mine segmentation takes 57.74 ms, and the end-to-end pipeline takes 558.83 ms. DBSCAN clustering is the largest remaining runtime bottleneck, and subsequent embedded deployment will prioritize optimized neighborhood search, parallel clustering, and validation on the target autonomous haulage system.

## Figures and Tables

**Table 1:** Analysis of the research gap and comparison of existing perception methods.

Method Category	Advantages	Limitations
**LiDAR-only**	High-precision geometry and resolution	Fails under occlusion; limited by LOS
Radar-only	All-weather and NLOS capabilities	Sparse data, high noise, and multipath interference
Deep Fusion	Powerful multimodal joint representation	Relies on overlapping observability; performance can degrade under complete asymmetric occlusion
Proposed (BSCF)	**Blind-spot complementarity via physical constraints**	**Requires calibrated spatio-temporal alignment**

**Table 2:** Comparison between VRR and commonly used perception metrics under NLOS occlusion.

Metric	Required Reference	Limitation Under NLOS Occlusion
IoU	Complete ground-truth box or occupancy region	Requires reliable full-object annotation, which is difficult when the target is invisible to LiDAR.
Recall	Target-level detection label	Indicates whether a target is detected, but does not quantify recovered spatial evidence.
Center error	Ground-truth target center	Evaluates localization accuracy but requires a reliable hidden-target center.
Occupancy recovery	Voxel-level occupancy ground truth	Requires dense voxel-level ground truth, which is difficult in large-scale mining scenes.
VRR	Reference bounding-envelope volume	Provides a proxy measure of partial envelope-level spatial evidence recovered from occluded regions.

**Table 3:** Detailed specifications of the sensors used in the experimental platform.

Parameter	LiDAR	4D mmWave Radar
Model	Innovusion Falcon Prime	AMDR405T2
Detection Range	500 m	300 m
FOV (Horizontal × Vertical)	120° × 25°	120° × 30°
Range Accuracy	±0.02 m	±0.03 m
Range Resolution	0.005 m	0.12 m
Angular Accuracy (Azimuth × Elevation)	±0.1° × ±0.1°	±0.1° × ±0.15°
Angular Resolution (Azimuth × Elevation)	0.09° × 0.08°	1.0° × 1.5°
Velocity Range	–	−120∼+60 m/s
Velocity Accuracy	–	±0.02 m/s
Velocity Resolution	–	0.05 m/s

**Table 4:** Design-level comparison between Patchwork-family ground segmentation methods.

Method	Scene Assumption	Main Mechanism	Design Limitation or Adaptation
Patchwork	Urban road with relatively regular ground	Concentric zone model and local plane fitting	Less suitable for large mining slopes and long-range height variation
Patchwork++	General outdoor LiDAR ground segmentation	Region-wise ground likelihood and adaptive criteria	Limited adaptation to open-pit unloading terrain
PatchWork-Mine	Unstructured mining road, slope, and unloading area	Mining-specific zone ranges, plane thresholds, and low-point filtering	Adapted to large-scale undulating terrain and residual radar-fusion distortions

**Table 5:** Detailed geometric feature information of the extracted obstacle instances.

ID	Manual Category	Volume (m^3^)	Number of Points	Centroid (x, y, z)
1	Soil slope	615.41	2792	[−12.73, 31.20, −1.54]
2	Road boundary	299.09	1142	[27.33, 28.93, −0.32]
3	Stepped bench	2095.67	491	[−33.19, 45.47, 4.55]
4	Road undulation	5.68	94	[−5.20, 68.03, −1.34]
5	Road undulation	0.56	22	[−21.61, 72.82, −1.92]
6	Road undulation	1.63	19	[43.83, 70.88, 1.51]
7	Occluding truck	762.41	3280	[−7.56, 96.07, 1.12]
8	Road boundary	0.92	43	[30.36, 93.54, −0.20]
9	Occluded truck	511.40	284	[−16.21, 113.47, 1.51]

**Table 6:** VRR comparison in the far-range (70 m) experiment. VRR1 denotes the front visible truck, and VRR2 denotes the rear occluded truck.

Method	VRR1 (First Truck)	VRR2 (Occluded Truck)
Radar-only	23.8%	18.2%
LiDAR-only	53.8%	5.0%
**Proposed (BSCF)**	**72.7%**	**59.3%**

**Table 7:** Comparison of Volume Recovery Rate (VRR) in the near-range (35 m) experiment.

Point Cloud Type	VRR1	VRR2	VRR3	VRR4
Fused Point Cloud	90.2%	15.6%	17.8%	18.7%
LiDAR Point Cloud	60.1%	–	8.7%	10.2%
Radar Point Cloud	36.7%	10.2%	6.9%	6.5%

**Table 8:** Geometric consistency metrics (CD and HD) in the real-world mining scenario. CD and HD are reported in meters.

Experiment	CD(F, L)	CD(R, L)	HD(F, L)	HD(R, L)
Far-range mining scene (70 m)	1.64	73.06	19.54 m	37.31 m
Near-range mining scene (35 m)	1.37	105.90	12.85 m	48.01 m

**Table 9:** Comparison of Volume Recovery Rate (VRR) in the test field experiment. VRR1 denotes the foreground haul truck, and VRR2 denotes the occluded steel shed.

Point Cloud Type	VRR1	VRR2
Fused Point Cloud	108.6%	5.2%
LiDAR Point Cloud	60.1%	–
Radar Point Cloud	104.1%	5.2%

**Table 10:** Geometric consistency metrics in the test field scenario. CD and HD are reported in meters.

Metrics	CD(F, L)	CD(R, L)	HD(F, L)	HD(R, L)
Value	1.62	27.82	21.55 m	27.51 m

**Table 11:** Scenario-wise point-count diagnostics of BSCF filtering variants in the far-range mining scene, near-range mining scene, and test-field extreme occlusion scene.

Scenario	Method	Injected Radar	Visible Leakage	Isolated Radar
Far-range 70 m	LiDAR-only	0	0	0
	Radar-only	5249	2737	16
	Direct fusion	5249	2737	16
	BSCF w/o spatial consistency	2512	0	18
	BSCF w/o blind-spot verification	5233	2733	**0**
	Full BSCF	**2500**	**0**	6
Near-range 35 m	LiDAR-only	0	0	0
	Radar-only	4825	1172	15
	Direct fusion	4825	1172	15
	BSCF w/o spatial consistency	3653	0	16
	BSCF w/o blind-spot verification	4810	1171	**1**
	Full BSCF	**3639**	**0**	3
Test field	LiDAR-only	0	0	0
	Radar-only	10,030	5745	2
	Direct fusion	10,030	5745	2
	BSCF w/o spatial consistency	4285	0	5
	BSCF w/o blind-spot verification	10,028	5745	**0**
	Full BSCF	**4283**	**0**	3

**Table 12:** Scene-level hidden-target radar cue counts based on manually associated mine-sequence ROIs.

Scenario	Method	Target ROI Cue	Visible Leakage
Far-range 70 m	LiDAR-only	0	0
	Direct fusion	358	2737
	Full BSCF	274	0
Near-range 35 m	LiDAR-only	0	0
	Direct fusion	209	1172
	Full BSCF	197	0

**Table 13:** Scenario -wise point-count ablation of radar preprocessing and blind-spot filtering for mine point-cloud sequences, including manually associated target-ROI cue counts.

Scene	Stage	Radar Points	Low-Z Ghosts	Isolated Radar	Visible Leakage	Target ROI Cue
mine1	Raw radar	7768	207	29	–	0
	RANSAC-filtered radar	7379	52	17	–	0
	Aligned filtered radar	7379	0	17	3239	371
	Spatial consistency only	7362	0	**2**	3236	371
	Full BSCF	**4126**	**0**	5	**0**	**306**
mine2	Raw radar	5502	50	23	–	0
	RANSAC-filtered radar	5249	0	16	–	0
	Aligned filtered radar	5249	0	16	2737	358
	Spatial consistency only	5233	0	**0**	2733	357
	Full BSCF	**2500**	**0**	6	**0**	**274**
mine3	Raw radar	5007	561	25	–	0
	RANSAC-filtered radar	4825	412	15	–	0
	Aligned filtered radar	4825	255	15	1172	209
	Spatial consistency only	4810	251	**1**	1171	207
	Full BSCF	**3639**	**173**	3	**0**	**197**

**Table 14:** Averaged module-level runtime summary measured in MATLAB R2024b.

Module	Mean ± Std.	Unit	Note
LiDAR preprocessing	32.58 ± 11.54	ms	Denoising and voxel downsampling of 40,227 LiDAR points
BSCF filtering module	39.23 ± 4.20	ms	Blind-spot complementary filtering of aligned radar and LiDAR points
PatchWork-Mine ground segmentation	57.74 ± 24.71	ms	Pure segmentation runtime without figure rendering or file saving
DBSCAN clustering	403.55 ± 21.49	ms	Dominant MATLAB-stage bottleneck for 10,605 non-ground points
Convex hull extraction	2.46 ± 1.41	ms	Obstacle-level geometric envelope extraction
End-to-end algorithm	558.83 ± 18.46	ms	Measured over 10 repeated runs on the mine1 frame

## Data Availability

Data are unavailable due to privacy restrictions. No public involvement or specific reporting guidelines were applicable. No AI or AI-assisted tools were used in drafting this manuscript.
